# Retraction: Dynamic changes of serum miR-105-3p expression and prognostic value evaluation of postoperative thyroid cancer

**DOI:** 10.3389/abp.2024.13857

**Published:** 2024-10-14

**Authors:** ABP Editorial Office

Following publication, concerns were raised about the duplicate publication of the article cited above in Acta Biochimica Polonica and Cellular and Molecular Biology [Zhou, J., Song X., Li Y. et al. (2023). Cell. Mol. Biol., 69. doi: 10.14715/cmb/2023.69.12.19].

An investigation was conducted in accordance with Acta Biochimica Polonica policies, whereby it was confirmed that the authors submitted the manuscript for consideration in two journals simultaneously.

Following the Committee on Publication Ethics guidelines, this article, published most recently, is retracted.

This retraction was approved by the Editor in Chief of Acta Biochimica Polonica. The authors received a communication regarding the retraction and had a chance to respond. This communication is recorded by the publisher.

